# Recent German Work on Tuberculosis

**Published:** 1916-11-25

**Authors:** 


### RECENT GERMAN WORK ON TUBERCULOSIS.

It seems ominous for the faith of believers in the treatment of tuberculosis by tuberculin that the country which furnished that substance is now attacking the therapeutic problem on different lines altogether—namely, by chemotherapy; in a word, on the lines of salvarsan. One enthusiast has even said that since 605 arsenic-containing compounds were tried against syphilis before success was obtained, so in like manner in the case of tuberculosis it is now only a question of trying a sufficient number of compounds of gold. Gold and copper, indeed, not arsenic, are the metals with which most anti-tuberculosis experiments are at present being conducted in Germany and Austria. But anti-tuberculosis laboratory experiments have been legion, and up to the present (if the aforesaid tuberculin true believers will excuse the remark) their practical therapeutic outcome has been truly exiguous. When one reads that *in vitro* a certain gold salt has shown itself "tropic" to the tubercle bacillus in almost infinitesimal dilution one feels still unmoved out of a scepticism which has been confirmed times without number. But when one reads, in a scantily distributed scientific publication which seems to lie outside all political influences, that the Lupus Sub-Committee of the German Central Committee for the combating of tuberculosis has erected a building, in connection with the municipal hospital at Barmen, for the purpose of treating lupic patients with a new copper compound, attention is naturally stirred. The substance in question is stated to be due to the prolonged work of Strauss (who has been given the direction of the institute alluded to), and to consist of a combination of copper with lecithin. Hence, perhaps, its name—lekutyl. Elsewhere (*Muenchener med. Wochenschrift*, 1916, s. 449) we are informed that lekutyl is composed of cinnamic acid, copper, and lecithin. Strauss has also written on the subject in the periodical *Strahlentherapie*. The medicament may be used both internally and externally, and it is recommended for consumption as well as for surgical tuberculosis. But by far its chief field of trial has been for lupus, when it is supplemented with heliotherapy of the artificial kind, by means of the mercury-vapour lamp.* Internal treatment is by means of pills, external by means of an ointment applied to the tuberculous focus. The latter is somewhat painful. The procedure is stated to be cheaper than *x*-ray applications, not dangerous, and more efficacious. The patient attends two or three times a week. Lekutyl is not as yet suitable for intravenous injection, but in lupus the results are stated (*Tuberculosis*, December 1915) to be becoming more and more recognised as undoubtedly highly favourable.

In Austria it is hoped that the claims of those invalided for tuberculosis brought about by military service will stimulate anti-tuberculosis measures after the war. The Austrian Central Anti-Tuberculosis Committee, in a memorial tendered to the Ministries of War, of the Interior, and of National Defence, which is published in the *Wiener klinische Wochenschrift*, 1916, Nr. 10, point out that the disease costs the country 160 million kröner yearly. In 1912 more than 81,000 people died of it; while after the war an increase in the mortality is to be expected. Not only does the strain of campaigning produce lung tuberculosis, but also injury conduces to surgical tubercle. (We believe, however, that shot wounds penetrating the lungs have not been at all often followed by consumption.) Therefore, all existing anti-tuberculosis agencies and institutions must be not only kept up but increased and enlarged, in order to afford every possible opportunity of cure or alleviation to war victims to this disease.

* This apparatus is, unfortunately, rare in this country; indeed we believe that that efficient institution, the Lord Mayor Treloar Cripples' Hospital at Alton, has the bulk of the lamps at present available for medical purposes.

